# Comparative analysis of correlation and causality inference in water quality problems with emphasis on TDS Karkheh River in Iran

**DOI:** 10.1038/s41598-025-85908-0

**Published:** 2025-01-22

**Authors:** Reza Shakeri, Hossein Amini, Farshid Fakheri, Man Yue Lam, Banafsheh Zahraie

**Affiliations:** 1 School of Civil Engineering, College of Engineering, University of Tehran, Tehran, Iran; 2 School of Engineering, Cardiff University, Cardiff, UK; 3 Department of Civil and Environmental Engineering, Amirkabir University of Technology, Tehran, Iran

**Keywords:** Water quality, Machine learning, Causality inference, Correlation, River, TDS, Environmental impact, Hydrology, Freshwater ecology

## Abstract

Water quality management is a critical aspect of environmental sustainability, particularly in arid and semi-arid regions such as Iran where water scarcity is compounded by quality degradation. This study delves into the causal relationships influencing water quality, focusing on Total Dissolved Solids (TDS) as a primary indicator in the Karkheh River, southwest Iran. Utilizing a comprehensive dataset spanning 50 years (1968–2018), this research integrates Machine Learning (ML) techniques to examine correlations and infer causality among multiple parameters, including flow rate (Q), Sodium (Na^+^), Magnesium (Mg^2+^), Calcium (Ca^2+^), Chloride (Cl^−^), Sulfate (SO_4_^2−^), Bicarbonates (HCO_3_^−^), and pH. For modeling the causation, the “Back door linear regression” approach has been considered which establishes a stable and interpretable framework in causal inference by focusing on clear assumptions. Predictive modeling was used to show the difference between correlation and causation along with interpretability modeling to make the predictive model transparent. Predictive modeling does not report the causality among the variables as it showed Mg is not contributing to the target (TDS) while the findings reveal that TDS is predominantly positive influenced by Mg, Na, Cl, Ca and SO_4_, with HCO_3_ and pH exerting negative (inverse) effects. Unlike correlations, causal relationships demonstrate directional and often unequal influences, highlighting Mg as a critical driver of TDS levels. This novel application of ML-based causal inference in water quality research provides a cost-effective and time-efficient alternative to traditional experimental methods. The results underscore the potential of ML-driven causal analysis to guide water resource management and policy-making. By identifying the key drivers of TDS, this study proposes targeted interventions to mitigate water quality deterioration. Moreover, the insights gained lay the foundation for developing early warning systems, ensuring proactive and sustainable water quality management in similar hydrological contexts.

## Introduction

Freshwater sources play a crucial role in maintaining the health and sustainable development of societies. Beyond mere quantity, the quality of water is equally important^1^. Ensuring safe access to drinking water yields tangible health benefits. Efforts should be directed toward achieving water safety as, major public health concern^2^, to the greatest extent possible (WHO 2011^92^). Surface waters, often scarce with high seasonality, face contamination challenges^3^. In recent decades, global warming, socio-economic growth, and population increase have intensified water resource utilization. Arid or semi-arid nations like Iran confront significant water quantity and quality issue^4–6^. Surface water serves as a vital global freshwater resource, and its deficiency or compromised quality can impact drinking water accessibility and environmental and economic development^7,8^.

Monitoring (river) water is essential for assessing its quality and pollution levels, which is vital for protecting both human health and the environment. Ensuring water quality requires ongoing surveillance of water resources^9^. Electrical Conductivity (EC) and Total Dissolved Solids (TDS) serve as vital indicators for assessing water quality. Elevated TDS levels have been linked to adverse health effects, including gastrointestinal distress and organ dysfunction. Similarly, discrepancies in EC values may signal the presence of harmful substances, prompting thorough investigation and remediation. These indicators play a pivotal role in safeguarding public health by upholding water purity standards^10–12^.

EC reflects the concentration of dissolved substances and minerals^7^. TDS encompass inorganic salts like sodium (Na^+1^), magnesium (Mg^+2^), calcium (Ca^+2^), and potassium (K ^+1^) as cations, along with chloride (Cl^−1^), sulfate (SO_4_^−2^), nitrates (NO^−3^), bicarbonates (HCO_3_^−1^) as anions, and other dissolved organic particles^13,14^. According to the World Health Organization (WHO) guidelines for water quality, acceptable TDS limits are 600 mg/l, while permissible limits are 1000 mg/l. Similarly, EC values should not exceed 500 µs/cm (acceptable) or 1500 µs/cm (permissible) to avoid adverse effects on human health.

Process-based models, such as numerical or analytical models, have been effective in simulating hydrodynamics and water quality in surface water systems^15,16^. However, their complexity in terms of application, data requirements, and simulation time, limits their use in managing drinking water sources^17–20^. Today, Machine Learning (ML) models as a subset of Artificial Intelligence (AI) have made significant strides in managing and monitoring water quality and hydrological processing^21^. ML algorithms analyze data to extract patterns and predict new information^22^. With the availability of large datasets, improved algorithms, and increased computing power, ML has gained widespread adoption in different fields like water and environmental science and engineering^23–28^. Its high accuracy, customization, and ease of development contribute to accurate assessment and prediction of complex environmental conditions, benefiting water resource management and water quality monitoring^29–38^.

Causal relationships in ML are very newfound feature within AI, requiring substantial research and development. Causal relations recognizes that mere correlations between variables are insufficient to establish causal relationships. While ML methods excel at predicting outcomes, they often fall short in understanding causality. Causality research can be broadly categorized into two main branches: causal discovery and causal inference. The former aims to extract causal knowledge directly from observational data, while the latter estimates the impact resulting from changes in specific variables on an outcome of interest^39^. Causal discovery methods have garnered significant attention in hydrometeorological research. Notably, they have been applied to understand the connections between different variables from observed data^40,41^. The four main categories of causal discovery methods include: Granger Causality (GC), Graph-Based Algorithms (e.g., the PC algorithm), Convergent Cross Mapping (CCM) and Structural Causal Models (SCM). These methods are well documented in the review study for climate research^42^ and hydrometeorological research^43^. Among these methods, GC stands out as the first practical approach for testing causality and, its widespread use in hydrometeorological research is attributed to its simplicity and strong performance in assessing causal interactions^44^. Causal inference models include linear and non-linear models in ML that typically rely on theory and prior knowledge to guide analyses, and it is not commonly associated with prediction modeling. However, modern causal inference methods, based on counterfactual or potential outcomes approaches, often involve intermediate processing steps before reaching the final estimation^45^. Previously mentioned, in the domain of exploration of causal relationships in ML, minimal research has been conducted across various scientific subjects, particularly in the context of water resources management and assessment (both quantitatively and qualitatively). Below, we highlight some of these studies.

Zhang et al.^46^, developed a grid-based interpretable ML approach to examine the spatial and temporal effects on water quality, focusing on nitrogen, phosphorus, and chemical oxygen demand in the Minjiang River Watershed, China. They discovered that reservoir water quality is more sensitive to environmental changes than stream water, with soil moisture and urbanization influencing pollutant distribution and point source pollution per inhabitant decreasing with urbanization. Han et al.^47^ examined the spatial and temporal changes in Na, K, Mg, Cl, SO_4_ and HCO_3_ in the Yellow River including. Their study identified nutrient sources and aided in forming strategies to control nutrient flow. The research divided the river into three regions showing varied ion and nutrient concentrations due to natural and human influences. Key findings revealed that phosphorus transport is rain erosion-driven, nitrogen sources vary seasonally, and six ions were identified as pollution tracers, enhancing hydro chemical analysis application in water management. Zavareh et al.^48^ delved into the connections among stream water quality indicators, hydroclimatic variables, and land characteristics to enhance water quality protection strategies. Principal Component and Granger causality analyses were applied to data gathered from ten eastern United States watersheds, revealing consistent patterns across locations and emphasizing the influence of factors like watershed size and land use on causal relationships. Mohammadi et al.^49^ investigated the relationship between water quality variables and catchment characteristics in Mazandaran Province, Iran. Their results revealed strong associations between variables like SAR, TDS, EC, Na, and Cl with geological features, while other variables showed connections with rainfall, land cover, and area.

In several previous studies, Adaptive Neuro-Fuzzy Inference System (ANFIS) demonstrated high efficiency in predicting water quality variables. For example, Karthikeyan et al.^50^ used Spearman’s rank correlations and found significant associations among pH, EC, Ca, HCO_3_, Mg, SO_4_, Na, K, and Cl, suggesting anthropogenic influences and sanitation issues impacting water quality. According to the result, Principal Component Analysis (PCA) highlighted strong relationships between Cl, K, Mg, dissolved solids, aiding in the assessment of hydro chemical characteristics in the Cauvery River, Tamil Nadu, Indial. Golekar et al.^51^ assessed the geochemical composition of surface and groundwater samples from 22 locations in Warnanagar, Maharashtra, India, focusing on key variables like pH, EC, and ion concentrations. Their results indicated elevated levels of salinity and hardness in the water, suggesting potential risks for drinking and irrigation purposes. He (2016) proposed a novel method for evaluating the causal relationship between water quality variables. In this method, a multivariate Gaussian mixture model was proposed to analyze joint distributions, providing insights into statistical dependence. Demonstrated using Bow River data in Canada, the approach offered a quantitative understanding of how variables influence each other in complex environments. Singh and Tripathi^52^ utilized Factor analysis to understand the relationships among hydro chemical variables in groundwater samples from NOIDA, part of the National Capital Region (NCR) of Delhi. Three factors, representing salinity, alkalinity, and pollution, were identified, explaining 79.3% of the total variance. The study emphasized importance of multivariate analysis in delineating areas affected by salinization, alkalinity, and pollution for effective groundwater management through techniques like rainwater harvesting and water softening. Bajpayee et al.^53^ employed Pearson correlation matrix and multiple linear regression analysis on 58 groundwater samples collected from the northeastern region of Bankura District, West Bengal, India, during pre and post monsoon seasons in 2009–2010. The analysis revealed strong correlations between TDS and EC with various groundwater constituents, indicating their significance in assessing water quality. Raju^54^ employed correlation and regression analyses to assess seasonal variations in well water quality parameters in the upper Gunjanaeru River basin, South India. Key findings indicated significant positive correlations between Specific Electrical Conductance (SEC) and most parameters, while multiple linear regression models highlighted the influence of variables like HCO_3_, SO_4_, and Cl on SEC during post- and pre-monsoon seasons. To date, a systematic causal inference study has not been conducted for the Karkheh River, Iran, that can be generalized to other rivers.

Researchers may now utilize these state-of-the-art techniques such as ML and Deep Learning (DL) to identify patterns in the dataset thanks to the significant advancements in ML algorithms and processing capacity^55,56^. There may be recurring patterns in any dataset that are repeatable and reproducible. One of the core concepts of AI is its capacity to reproduce patterns from a dataset without explicit instructions^57,58^. Giving the causal inference elaboration using the relevant examples, in most cases, the goal of causal inference in water quality research is examining how a given outcome is impacted by the quality of the water. For instance, there are two separate sources of water, and their chemical compositions are different. While the other source has a lower pH and a higher concentration of SO_4_, the first source has a higher pH level and a lower concentration of SO_4_. TDS, SO_4_ and pH concentrations are tested in each water source in order to determine how these water quality factors affect the final result. This implies that the variables of water quality (pH and SO_4_) and the result (TDS) may be causally related^59^. In this work, as it has been mentioned earlier, we conduct causal inference analysis to understand the causal relationship between the variables and TDS as an important and widely used and measured water quality indicator. To this end, it is not a matter of using empirical or experimental methodologies but rather, we use advance ML algorithms to have insight into this causation between variables and the outcome which in this case is TDS. By our proposed methodology, instead of running time-consuming and expensive experiments, we are going to show it is possible to use AI to go through the collected data and try to find the causation. This way of employing ML is new in the field and provides a new horizon to water quality data assessment and policy development. In addition to the introduction of a new methodology for understanding the causal relationship between the variables in water quality application, the novelty of this study lies in different reasons. For instance, it provides the knowledge-based to design the mitigation strategies by policy and decision makers based on the water quality condition through answering “What-if” questions. As an example, the question like “What would happen to TDS if Mg level reduced?” Also, it can be integrated with other fields such as socio-economic fields. Another important message of this work is the emphasis on the differences between causation and correlation by illustrating how they can be mistakenly used interchangeably. On the other hand, the case study chosen for this research, is located in semi-arid area with variability water flow and water scarcity across the region which makes it good to be exemplified in other similar regions. Facing the “Bias” is highly crucial in a data-based analysis. To this end, data scientists and researchers in fields where ML algorithms are applicable have sought to develop strategies to mitigate overfitting and bias. One of the strategies, is the interpretability modeling and cross check the results with domain expertise and the literature to ensure about the results. In this study, these two approaches have been taken into consideration.

## Material and methods

### Study area

A prevalent challenge encountered in hydro-environmental research is the scarcity of consistent and pertinent data. To circumvent this limitation, the Karkheh River was meticulously chosen as the focal point of this investigation due to its extensive data record spanning over half a century. This lengthy dataset is indispensable for comprehensive studies in this field. Moreover, the Karkheh River presents an apt case for examining the implications of fluctuating historical water quality conditions influenced by climate change, urban development, and anthropogenic activities^60,61^.

**Fig. 1:**
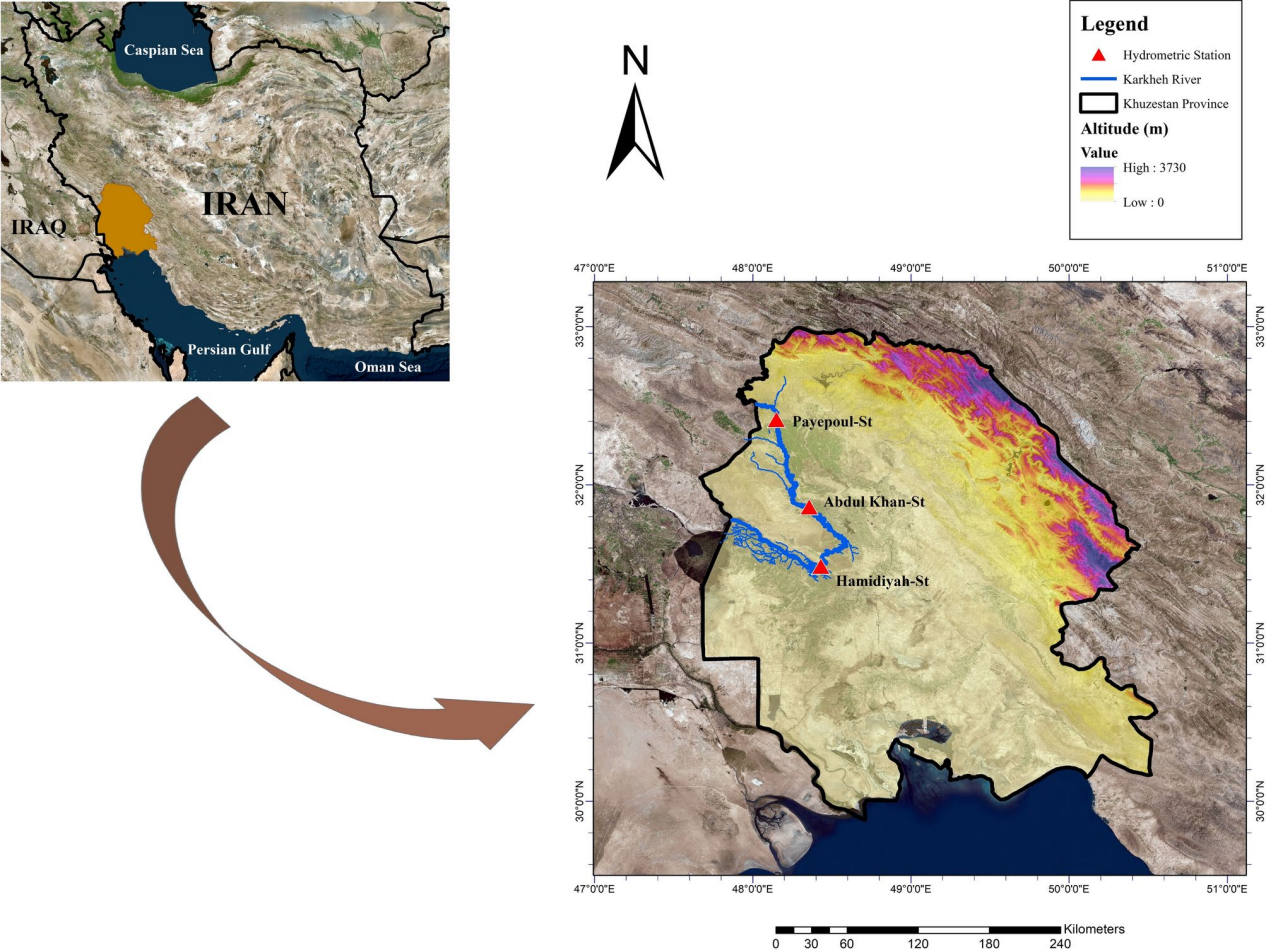
Study area and drainage network of the Karkheh River in Khuzestan Province.

Stretching approximately 950 km, the Karkheh River ranks as the third longest river in Iran, coursing from the western to southwestern regions of the nation. Originating from the Zagros Mountains in western Iran, the river eventually empties into the Persian Gulf via the Hurolazim wetland, situated at the Iran-Iraq border. In a predominantly arid to semi-arid climatic zone, the Karkheh River, with an average flow rate of 177.8 m^3^/s^62,63^ and an expansive catchment area of approximately 51,912.3 Km^2^, holds significant hydrological importance. This riverine system is characterized by a climate marked by chilly winters and protracted, scorching summers. The region receives an average annual precipitation of close to 360 mm. Concurrently, the average annual evaporation in the vicinity of the river reaches approximately 3200 mm. The climatic conditions are further defined by an average temperature of 19 °C and a relative humidity level averaging at 37% from the early 1990s to around 2020^64^. Elevational gradients along the Karkheh River are notably diverse, ranging from an altitude of about 3000 m above sea level down to 500 m^65^. Socio-economically, the Karkheh River exerts a considerable influence on the regions it traverses, catering to various needs such as irrigation, potable water supply, and industrial applications. The geographical scope of this study is illustrated in Fig. 1, which delineates the study area’s location and the trajectory of the Karkheh River within Khuzestan province^66^.

### Dara preparation

Data pertaining to both qualitative and quantitative parameters gathered in three hydrometric stations on Karkheh River has been obtained from the Iran Water Resources Management Company. These data were sampled monthly during 1968–2018. These stations are strategically located at proper distances along the river:

- Payepol station at coordinates 48° 08′ E and 32° 24′ N.
- Abdul Khan station at coordinates 48° 21′ E and 31° 51′ N.
- Hamidiyeh station at coordinates 48° 25′ E and 31° 29′ N.

To address data gaps, interpolation techniques were applied using the nearest time periods of measured values (employing a linear regression). Additionally, statistical methods from ML were used to assess the normal distribution of all data. Table 1 presents the quality parameters of the Karkheh River, comparing them to the acceptable and permissible values according to WHO guidelines. The data for this study has been provided by the local authorities that can be made available upon request.

**Table 1:** The long-term statistics of the monthly water quality parameters of the Karkheh River compared to the WHO standard values for drinking.

Parameter	Value					
	Average of three stations				Max acceptable value (WHO)	Max allowed value (WHO)
	Min.	Max.	Mean	Standard Deviation		
TDS (mg/l)	344.3	1601	857.2	231.5	600	1000
EC (µs/cm)	613	2446	1316.4	360.1	500	1500
Na (mg/l)	1.2	14.2	5.6	2.5	200	
Mg (mg/l)	1	5	2.7	0.74	50	150
Ca (mg/l)	2.5	12.5	5	1.3	75	200
pH (µs/cm)	5.3	8.5	7.9	0.26	6.5–8.5	
Cl (mg/l)	0.95	14.7	5.5	2.5	200	600
SO_4_ (mg/l)	1.7	11.7	5.1	1.7	200	400
HCO_3_ (mg/l)	1.1	4	2.7	0.43	150	

### Methodology

**Fig. 2:**
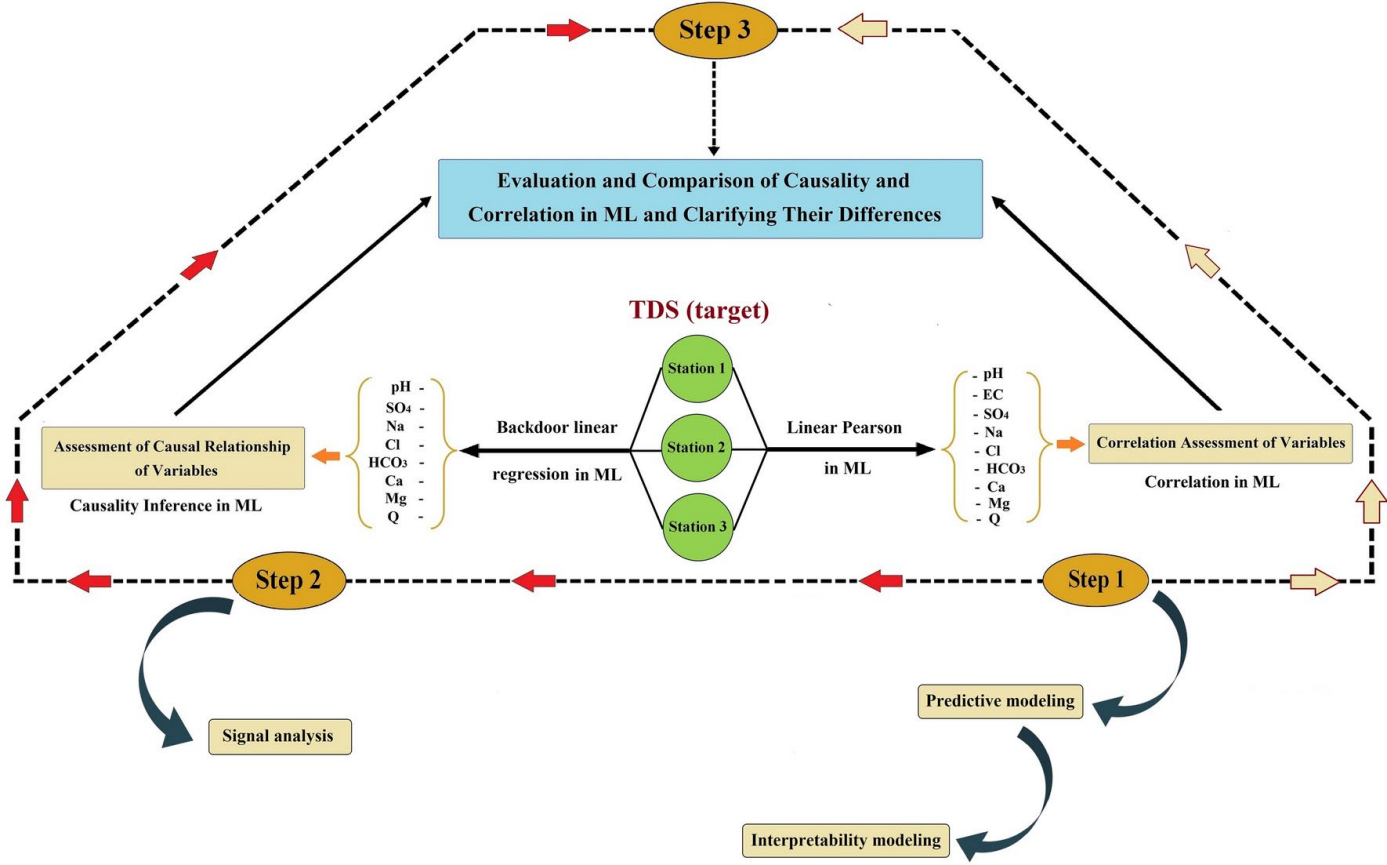
Flowchart of the research methodology.

This study aims at showing the differences between the causation and correlation. The first step is using Pearson coefficient correlation and using an interpretable predictive algorithm to see the contributions of the variables in the prediction. The next step, is using causal inference modeling (in this case, “back door linear regression”) to realize the causal effect of different water quality stressor. The last step is comparing the results between correlation-based and predictive modeling, and a causal modeling. The research flowchart is presented in Fig. 2.

### Signal analysis

In the context of causal inference, “Temporal dynamics” makes an important contribution to the causal relationships between variables and the outcome^67–70^. It is important to check whether or not, the temporal dynamics exist and if so, how stable the signal is. This analysis illustrates the necessity of using non-linear causal model or linear causal model. For this study, as it has been mentioned before, the causal method does not inherently and explicitly consider the temporal dynamics however it is important to make sure that the outcome would be meaningful. To address this, continuous wavelet transform has been employed to check if there is a significant temporal dynamic in the variables. In wavelet transform analysis, there are two types of waves: Father waves and Mother waves. Whereas the second part is the mother part of the decomposition, which is useful for comprehending the signals’ details, the first part is the father wave and is good at capturing the approximate smoothness of the signal, even at low frequencies^71–74^ The result of this decomposition is “Trend”, “Seasonality”, and “Residual”. For this study, to check the decomposition condition, “Haar” wavelet was considered. In principle, Haar wavelet, that is a mother wavelet, has capability of capturing the details of the signals since, unlike other wavelets that can be difficult to compute, the Haar wavelet is easy to generate and can be prepared using basic arithmetic operations^75,76^. The Haar wavelet’s multi-scale lossless rapid signal decomposition is its primary benefit^77^. This approach generates a general framework for the future studies using sophisticated signal analysis.

### Causality inference and correlation in ML

In a simple form of understanding the causal inference, it aims for discovering the causal-effect relationships between variables. In this context, the main goal of ML algorithm is not to predict the outcomes but to identify how changing one variable would cause a change in the outcome or another variable. For the sake of clarity, let assume *X* is the effect when *Y* happens so in structural equation modelling or graph-based models $Y= {\beta }_{0}+{\beta }_{1}X+\varepsilon$ where ${\beta }_{1}$ is the causal effect of *X* on *Y*^78^. Also, in terms of confounding effect, one variable might not affect directly on the outcome but a combination of that variable with another variable might make a new variable that may significantly contributes to the outcome. Exploring this confounding effect is also one of the aims of studying causality and causal inference. In a mathematical form $Y= {\beta }_{0}+{\beta }_{1}X+{\beta }_{2}Z+\varepsilon$ where *Z* can bias the estimate of the causal effect. For example, pH and HCO_3_, and Mg might affect the concentration of TDS so mathematically we need to adjust the model.

The complex relationship between different chemical components and water pollution selected indicators (TDS/EC in this study) and the climate change is still unknown, even after previous studies using different approaches^79–82^, For decades, the causal linkage between those components (variables) and water quality indices in rivers has drawn researchers’ attention^81,83^. Employing both experimental methodologies, empirical studies and numerical modelling, yet they continue to explore the complexities behind the water quality indices, pollution initiation and development, and how other big influencers such as climate change causally affect them^84,85^. While experiments offer tight control, guaranteeing internal validities, they inherently lack of comprehensive representation of natural systems^86^. In these environments, confounding variables and counterfactuals, the interplay of which cannot be fully captured in the experiments, emerge as significant limitations^87^. To untangle this complexity, this study suggests using data-driven models which in this case is the causal inference model. ML algorithms and causal inference techniques, in particular offer a powerful alternative, enabling the simultaneous investigation of all relevant factors, including confounders and counterfactuals that would be prohibitively time-consuming or expensive to explore experimentally^88^.

Correlation and causality inference may be considered the same or used interchangeably, which is a mistake. Correlation assessment has also been done using Pearson correlation coefficient.

1 $$\rho_{XY} = \frac{COV(X,Y)}{{\sigma_{X} \sigma_{Y} }}$$

where ${\rho }_{XY}$ is the Pearson’s coefficient, $COV\left(X,Y\right)$ is the covariance between two generic variables *X* and *Y*, and ${\sigma }$ is standard deviation of the two variables. The value of correlation coefficient lies between − 1 and + 1. Values near + 1 indicate the presence of a strong positive relation between variables, whereas the values approaching − 1 illustrate strong negative relation between variables^19^. In this work, we have taken steps further as it is obvious there is a correlation between water quality variables. This study aims at understanding how the selected water quality variables, individually or together, causally influences the target variable (TDS) and each other.

In order to deviate from traditional statistical analysis toward causal analysis of multivariate data, a paradigmatic shift needs to be made, as this study attempts to emphasize. The assumptions underlying all causal inferences, the languages employed to express those assumptions, the conditionality of all causal and counterfactual claims, and the techniques devised for evaluating them are all given particular attention. This study’s specific objective is to better understand the causative mechanism underlying how chemical component descriptors affect TDS in water by applying ML models and causal inference techniques. There are different causal inference approaches like structure-based causal inference modelling or graph-based modelling. For this study, we use Directed Acyclic Graphs (DAG), as a graph-based causal model, to discuss the causal paths.

The model has been employed is called “Back Door Linear Regression” that primarily relies on satisfying the backdoor criterion to estimate causal inference. This method highlights that the backdoor linear regression assumes static causal relationships. In other words, this method identifies how many sets of variables need to be adjusted in order to identify causal effects from observational data. Also, this model, establishes a stable and interpretable framework to understand the causal relationship between variables such as pH, HCO_3_, Mg, Na on the outcome, which is TDS without making the problem complex to be able to analyse. This is the first step in comprehending the causal effect. The insights gained though this study provides a foundation for exploring application of more advanced causal modelling techniques. Also, the models remove that spurious association, ensuring that the causal effect that is estimated is a true causal relationship. In the model employed for identifying the potential causal relationships among the variables, first the causal graph was discovered using causal graph visualization. An iterative analysis was adopted over each feature (pH, HCO_3_, Cl, SO_4_, K, Ca, Mg, Na) to find causal influences on the outcome which is TDS here. This method aligns with the causal discovery methods that systematically examine the causal relationships. Then the causal effect was estimated using linear regression and finally the causal effect was calculated. In this method (which has been developed using DoWhy library) the causal model assumes a linear relationship of the form:

2 $$Y \, = \alpha + \beta X + \varepsilon$$

where *Y* is the dependent variable, *X* is the independent variable and *α* and *β* are the parameters to be estimated and *ε* is the error term. To elaborate on the equation, X has a causal effect on Y, represented by β which captures how much *Y* changes when *X* changes. Statistical inference was done under two assumptions: Confidence intervals, that provide a range within which the true value of *β* is likely to lie, and Test Stat Significance which involves hypothesis testing to determine whether the estimated *β* is statistically significantly different from zero.

There are key assumptions in this method that have been taken into considerations:

- Linearity: The approach assumes a linear relationship between treatment and the outcome.
- Causal interpretation: The estimated *β* is interpreted as the causal effect under the assumption that the specified causal model correctly identifies and adjusts for confounders.
- Adjustment for the confounders: The effectiveness of the causal inference depends on the correctly specifying and adjusting for the confounding variables.

### Interpretability modeling

As it was mentioned earlier, in order to avoid the potential bias, there are different approaches like using interpretability modeling or the causal method that also inherently is interpretable. We have used interpretability modeling separately which enlightens the contribution of each variable in the target, however, the back door linear egression, that has been used, has an interpretable result due to the nature of the method, and the interpretability here has just been used to show the differences between the predictive models compared to causal models. To this end, the SAHP value method has been taken into consideration. This method can make the process inside the model (black box) transparent for understanding the variables contributions to the predictions. This will be helpful for assisting policymakers in ensuring that mitigation mechanisms are in place. The SAHP value for each feature indicates its average marginal contribution to the prediction, considering the order in which it is added the influence of other features^89–91^. The simplified representation of SAHP value is written bellow:

3 $$SHAP\left(feature\right)=average (all coalitions) \{weight \left(coalition\right)\times marginal contribution (feature, coalition)\}$$

where “*coalition*” refers to a specific combination of features (variables) in a dataset. In the context of SAHP value, the concept of coalition from game theory, in which, coalition means a group of players that are collaborating with each other to reach the target. In order to produce the result regarding the SAHP value and interpretability in predictive modelling, a well know machine learning model called “XGBoost” which is an ensemble learning algorithm, was employed. The model was used to predict the TDS level and then SHAP value was used to see the importance of each variable in the prediction. To elaborate on the XGBoost model, it is gradient boosting model that tries to find the local optima through boosting regression using different criterion. The model was just employed to have the interpretability results from a predictive model.

## Results and discussion

In this section we present the results which match state of the art methods. Here we compared the differences between terminologies of correlation and causation which enlightens the importance of using this new methodology for further investigations in the field. Planned comparison revealed how different these two are. A novel finding is the use of this methodology for policy makers to decided how to mitigate environmental crisis in future based on the available data and key variables to design an early warning system. For the analysis which will be presented in this part, the outcome was TDS and the treatments were the other input variables such as HCO_3_, Cl, SO_4_, K, Ca, Mg, Na. These treatments also were considered as outcome for other treatments to see how they may influence each other. What is presented in this work does not mean that there is no need for the experimental works as they are essential and vital for developments yet expensive and timely demanding, but rather, this work suggests another way of realizing the factors’ influences to design a new pathway for the future studies and finding the right directions. Since this is very important to make sure that the causal pathways which will be found out through causal inference are consistent with experiments, it is vital to ensure about the validation and statistical robustness of the results. The causal effect values have been checked using *P*-value in addition to the experimental literatures that have shown the causal pathways of different variables on the TDS level.

### Signal decomposition

The main aim of this study is to establish a baseline understanding about causal relationship between the variables without making the whole study complicated. Thus, it is essential to ensure about the temporal dynamics through seasonality, trend and the residual to make sure if there is temporal dynamics and if there is, it does not causally affect the outcome. Although there seems to have a long-term trend and seasonal variation in data but the residuals from the decomposition appear relatively stable across the time, which indicates while temporal dynamics exists, but the relationship can still be meaningfully approximated using back door linear regression at this stage.

**Fig. 3:**
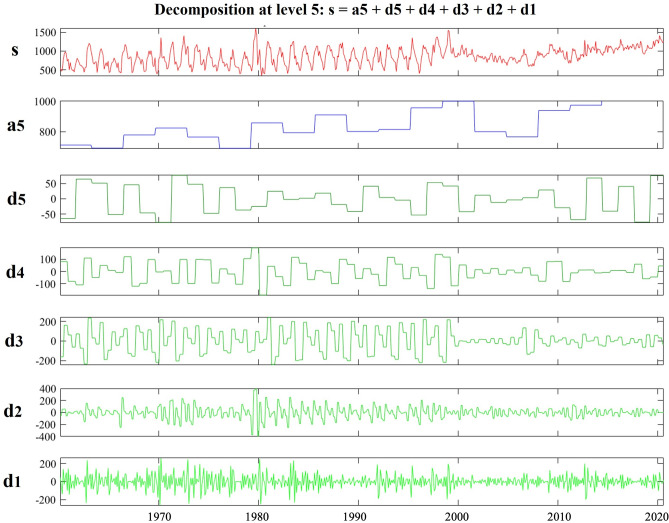
Signal analysis of TDS (s) using Haar wavelet with 5 levels of details (d_1_, …, d_5_) and approximation (a_5_).

Figure 3 shows analysis of TDS signals using Haar wavelets, focusing on five levels of detail and approximation, and “s” represents the real signal, a_5_ refers the approximation, and d_1_ to d_5_ are the details of the decomposition. Decomposing means that, it is possible to reconstruct the signal using approximation(s) and details. As can be seen in this figure, because of smaller magnitudes, the short-term variations (higher-frequency components or residuals) are less likely to significantly alter the overall causal links. In other words, signal decomposition illustrates that the trend is comparatively steady and smooth. As it is shown, although it shows the existence of some level of temporal dynamics, but it is enough stable to use linear regression for this stage.

### Interpretability on the predictive modeling

**Fig. 4:**
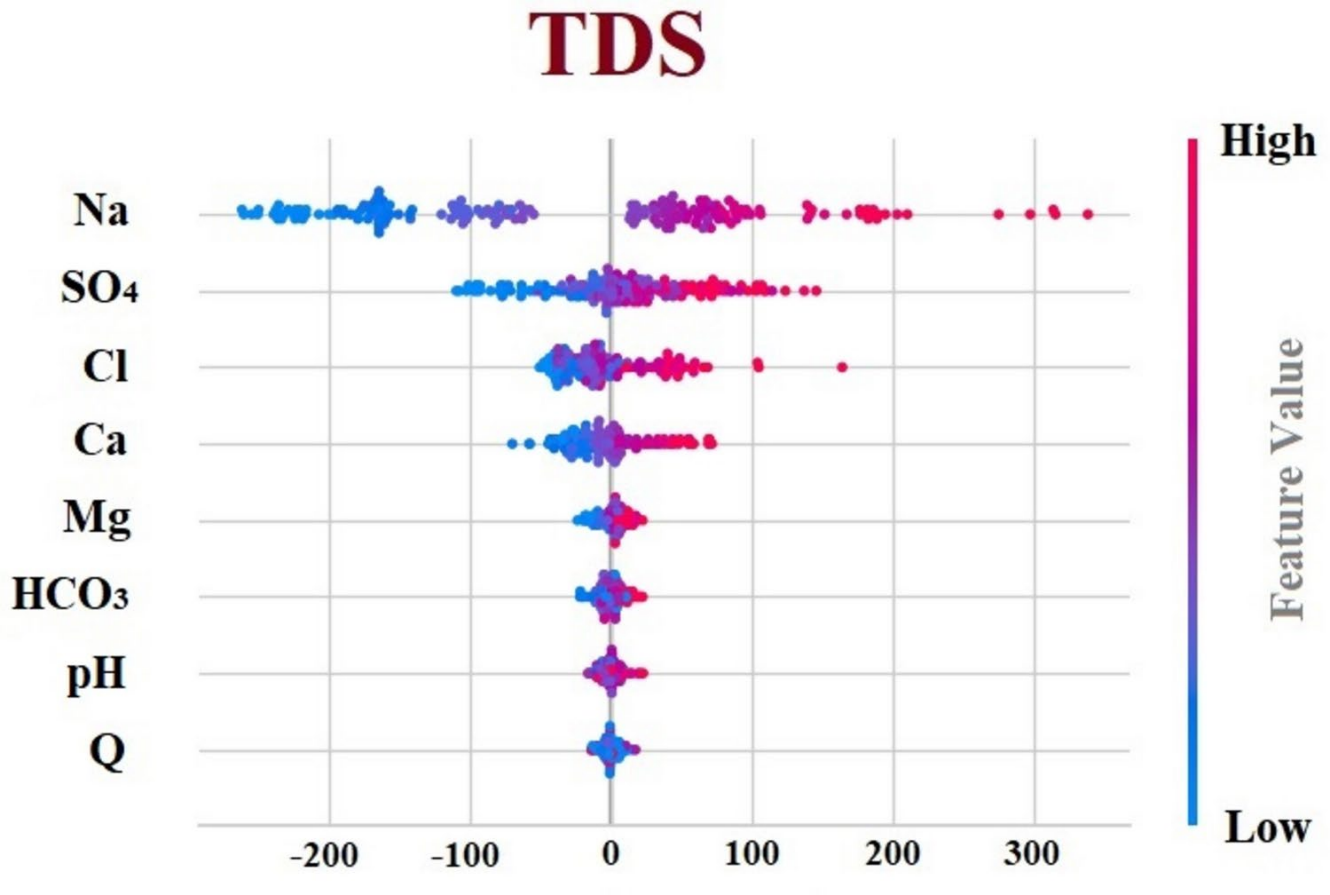
SHAP value for the predictive modeling based on XGBoost algorithm.

As it was mentioned earlier, the predictive models are meant for the prediction of the target which in this case is TDS level. This prediction is done through data analysis and the correlation that exists among variables or pair of variables that in turn causes to have the prediction with different accuracy. To show the differences between the predictive modeling and causation modeling, Fig. 4 shows the importance and contribution of each variable to the target. To read the SHAP value figures, the middle column and the scarcity are the main important items to be considered. The more variable is scattered compared to the middle column, the significant it is and vice versa, the more stick to the middle column, less significant contribute to the prediction. As can be in the figure, Na, SO_4_, Cl, and Ca respectively shows the most significant contribution to the prediction and the four variables—Mg, HCO_3_, pH, and Q—do not significantly affect TDS.

### Causality and correlation mapping

#### Payepol station

**Fig. 5:**
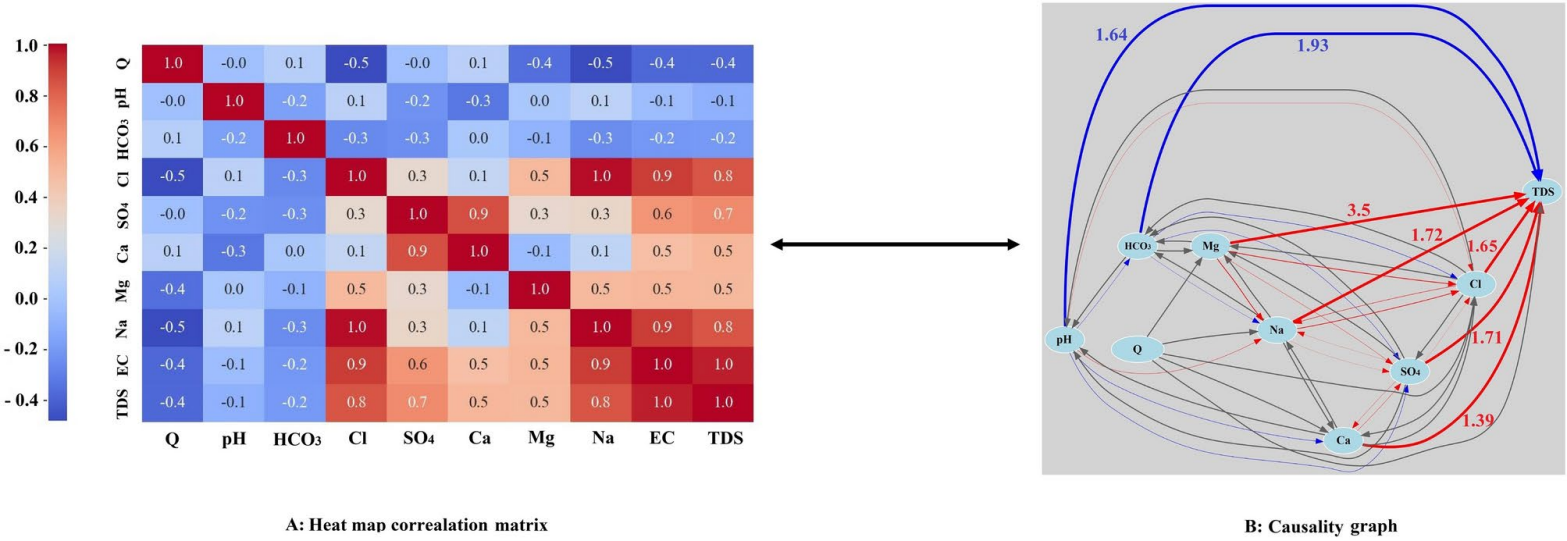
(**A**) Correlation heat map matrix and (**B**) causality map in the Payepol station (red lines: positive effect, blue lines: negative effect, gray lines: insignificant effects, thicker lines: stronger effects).

In Fig. 5, we present correlation and causality maps for various variables in relation to TDS and each other, based on linear relationships observed at the Payepol station. According to the figure, the following variables exhibit the highest positive correlation with TDS:

- Na with a correlation coefficient of + 0.8.
- Cl with a correlation coefficient of + 0.8.
- SO_4_ with a correlation coefficient of + 0.7.
- Ca with a correlation coefficient of + 0.5.
- Mg with a correlation coefficient of + 0.5.

Conversely, the following variables show an inverse and weaker correlation (compared to positive values) with TDS:

- Q with a correlation coefficient of − 0.4.
- HCO_3_ with a correlation coefficient of − 0.2.
- pH with a correlation coefficient of − 0.1.

The same trends are evident in the causality graph. Among the positively correlated variables, Mg (+ 3.5), Na (+ 1.72), SO_4_ (+ 1.71), Cl (+ 1.65) and Ca (+ 1.39) have the greatest impact on TDS. HCO_3_ (− 1.93) and pH (− 1.64) have the most negative impact on TDS.

The correlation between variables is bidirectional, but it does not necessarily imply causality. Let’s consider an example: the correlation coefficient between Mg and TDS is + 0.5. Similarly, the correlation between TDS and Mg is also + 0.5. However, when we examine the causality graph, it becomes evident that TDS is influenced by the amount of Mg, but the reverse relationship is not necessarily true. This apparent contradiction can be understood by considering the definition of TDS, which represents the sum of various cations and anions. Intuitively, one might expect that TDS is caused by Mg. However, exploring the relationships among other variables (excluding TDS) and their mutual influences is a complex task that often requires laboratory experience. In such cases, causal relationships become crucial and can partially replace experimental relationships. For instance, let’s look at the correlation coefficient between Mg and Cl, which is + 0.5. The causality graph reveals that Mg has a positive effect on Cl, but Cl has only a negligible effect on Mg. Similarly, pH negatively affects HCO_3_, but the reverse effect is insignificant (with an R^2^ value of − 0.2). The remaining variables correlation and causal relationships can be explored in Fig. 5. Examining causality goes beyond assessing correlation strength, and the key distinction lies in understanding how variables relate to each other. In essence, this work aims to uncover the unique and specific causal relationships among qualitative variables (especially variables other than TDS). Karthikeyan et al.^50^ experimentally illustrated that Mg has caused an increase in the TDS level which proves the causal pathway that has been identified through causal inference model.

**Fig. 6:**
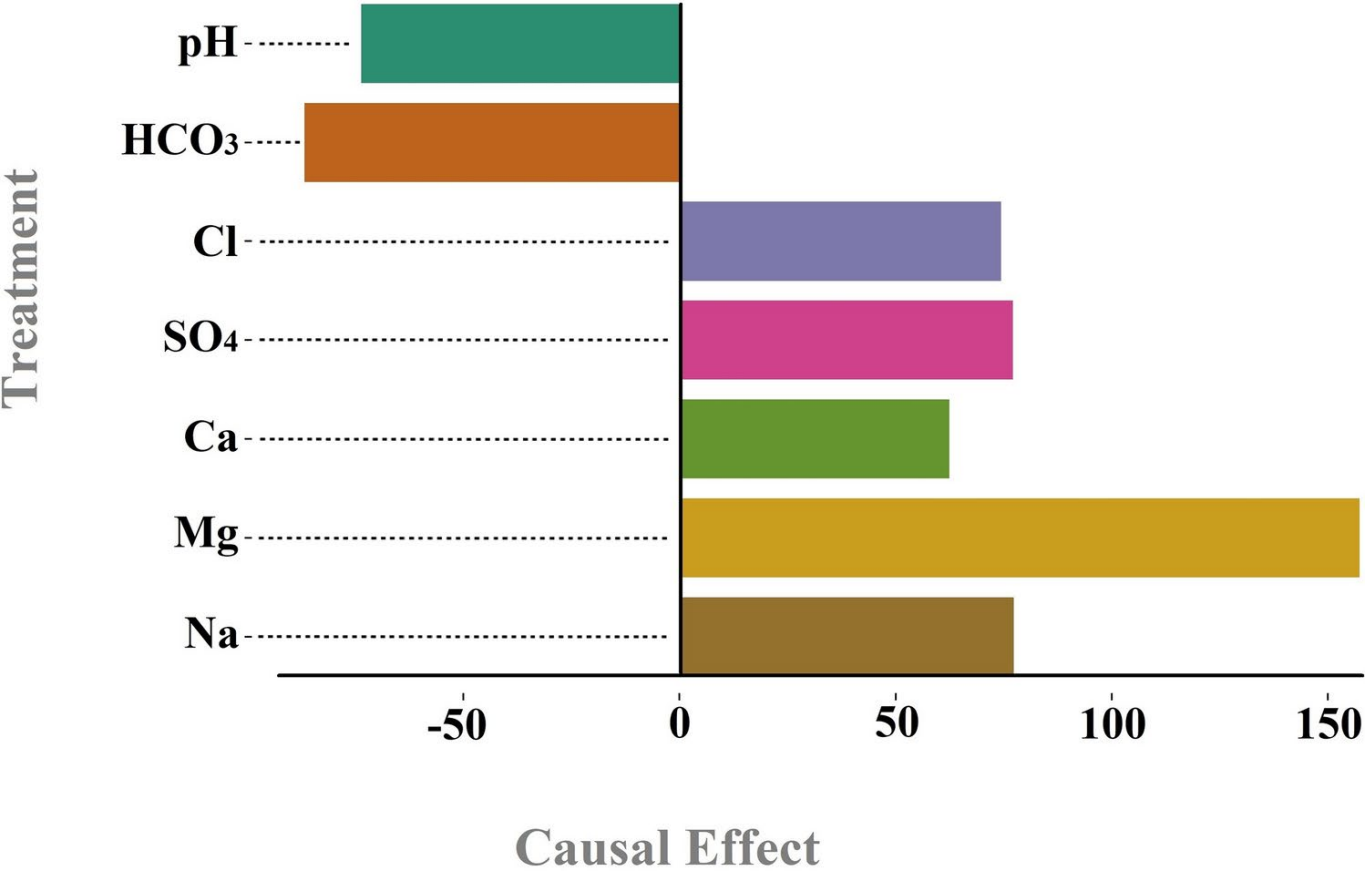
The influence of the examined variables on TDS in the Payepol station.

The impact of the analyzed variables on TDS is illustrated in Fig. 6 below. According to this figure, Mg, Na, SO_4_, Cl, and Ca have the most positive influence on TDS, while HCO_3_ and pH have the most negative influence on TDS, respectively. As it was proven in the chemical literature, we were expecting to see Mg key role in determining TDS condition. From the casual graph shown in the Figs. 5 and 6, in Payepol station, Mg play a critical role in influencing TDS. Our results by causal inference in ML cast a new light on the way of understanding how influential factors are affecting the outcome. As it has been mentioned and it was shown in the Fig. 6, Mg causally influences an increase in the TDS level while it was not seen in the SHAP value (Fig. 4) as an important role player in the prediction.

#### Abdul Khan station

**Fig. 7:**
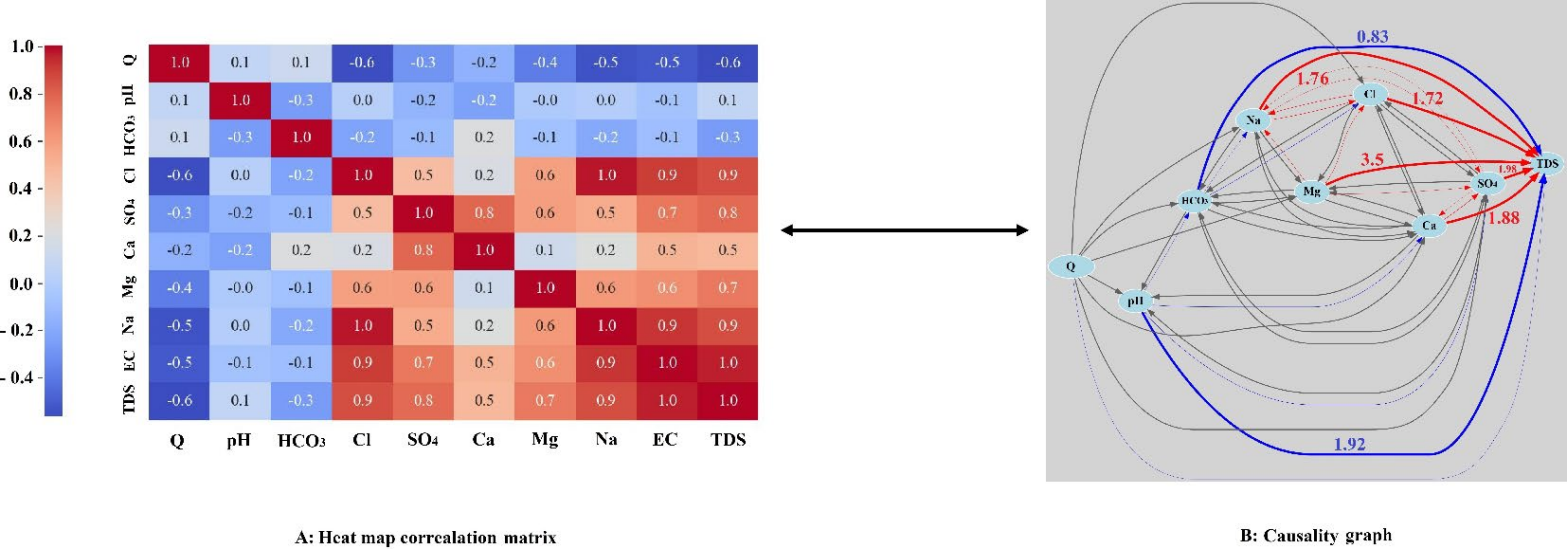
Correlation heat map matrix (**A**) and causality map (**B**) in the Abdul Khan station.

Figure 7 illustrates the heat map matrix and causal relationship map for the Abdul Khan station. Accordingly, TDS has a strong positive correlation with Na (+ 0.9), highlighting that sodium is a significant contributor to the TDS in the water. Similarly, the correlation between TDS and Cl is also notably high (+ 0.9), indicating that chloride ions are major constituents of the dissolved solids. There is a significant positive correlation (+ 0.8) between TDS and SO_4_, suggesting that sulfate ions are prevalent in the dissolved solids measured in the water. The variables pH (+ 0.1), Ca (+ 0.5), and Mg (+ 0.7) exhibit the weakest positive correlations with TDS. Conversely, the variables Q and HCO_3_ show the strongest inverse correlations with TDS, with R^2^ values of − 0.6 and − 0.3, respectively. Other notable correlations include a strong positive relationship between Na and Cl (+ 1.0), Na and SO_4_ (+ 0.8), and Cl and SO_4_ (+ 0.6). These correlations imply that these ions often coexist in the water, contributing collectively to the TDS.

The causality graph provides a more nuanced understanding of the directional influence among variables. It allows us to discern not just the correlation but the causative impact of one variable on another. The graph indicates several direct and indirect pathways influencing TDS. Significant causative relationships are observed from Mg (+ 3.5), SO_4_ (+ 1.98), Ca (+ 1.88), Na (+ 1.76), and Cl (+ 1.72) to the TDS, respectively, highlighting these ions as major causal agents in determining the TDS levels. Additionally, the variables pH (− 1.92) and HCO_3_ (− 0.83) negatively impact TDS. The causal graph illustrates both bidirectional and unidirectional relationships among variables, with some examples highlighted. While all variables influence TDS, the reverse is not true. Additionally, Mg has a stronger positive effect on Na than Na has on Mg, despite both having a correlation coefficient of + 0.6. Although R^2^ between pH and TDS is + 0.1, the causality graph indicates that pH significantly negatively impacts TDS. Similarly, HCO_3_ negatively affects Na, but the reverse effect is weaker. The remaining relationships and correlation coefficients are depicted in Fig. 7.

**Fig. 8:**
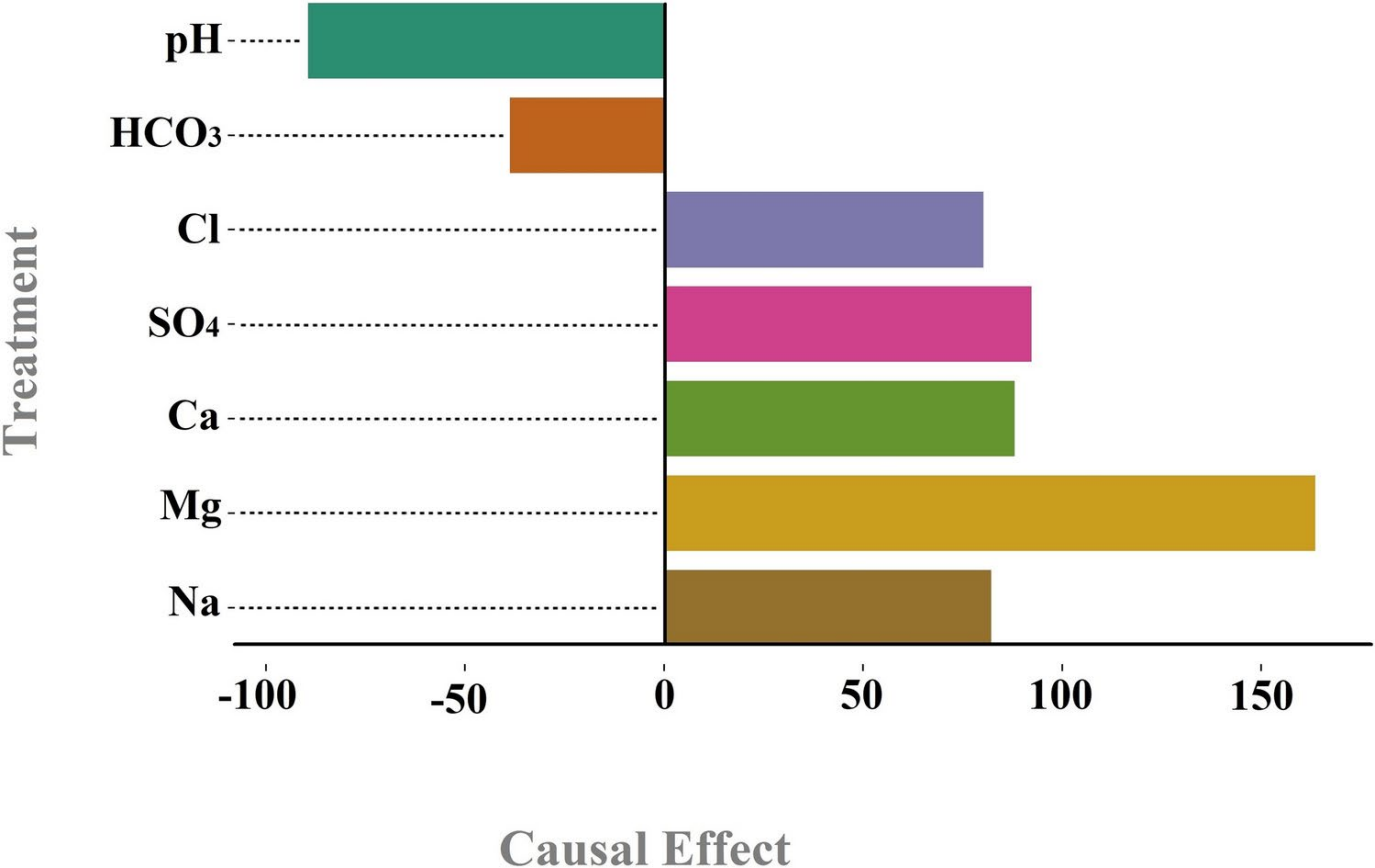
The influence of the examined variables on TDS in the Abdul Khan station.

Figure 8 illustrates the influence of the studied variables on TDS. Among these, Mg, SO_4_, Ca, Na, and Cl exhibit the most positive impact, respectively, with Mg being nearly twice as effective as the other four variables, which have similar effects. Conversely, pH and HCO_3_ negatively affect TDS. According to the figures in causal graphs of two mentioned stations (Figs. 6 and 8), the values were normalized with respect to the highest value which was Mg as it was mentioned earlier, it was expected. Therefore, the values shown in the causal graph are saying how important the factor is with respect to the other treatments. Also, in order to be statistically on the safe side, a control group of the outcome has been taken into consideration. In the sense that outcome when it gets treatment has been compared with the time that it has not received treatment and then the average treatment effect has been computed. Golekar et al.^51^, through the experimental work, showed the significance of the Mg level on the TDS level which is seen in the Fig. 8.

### Hamidiyeh station

**Fig. 9:**
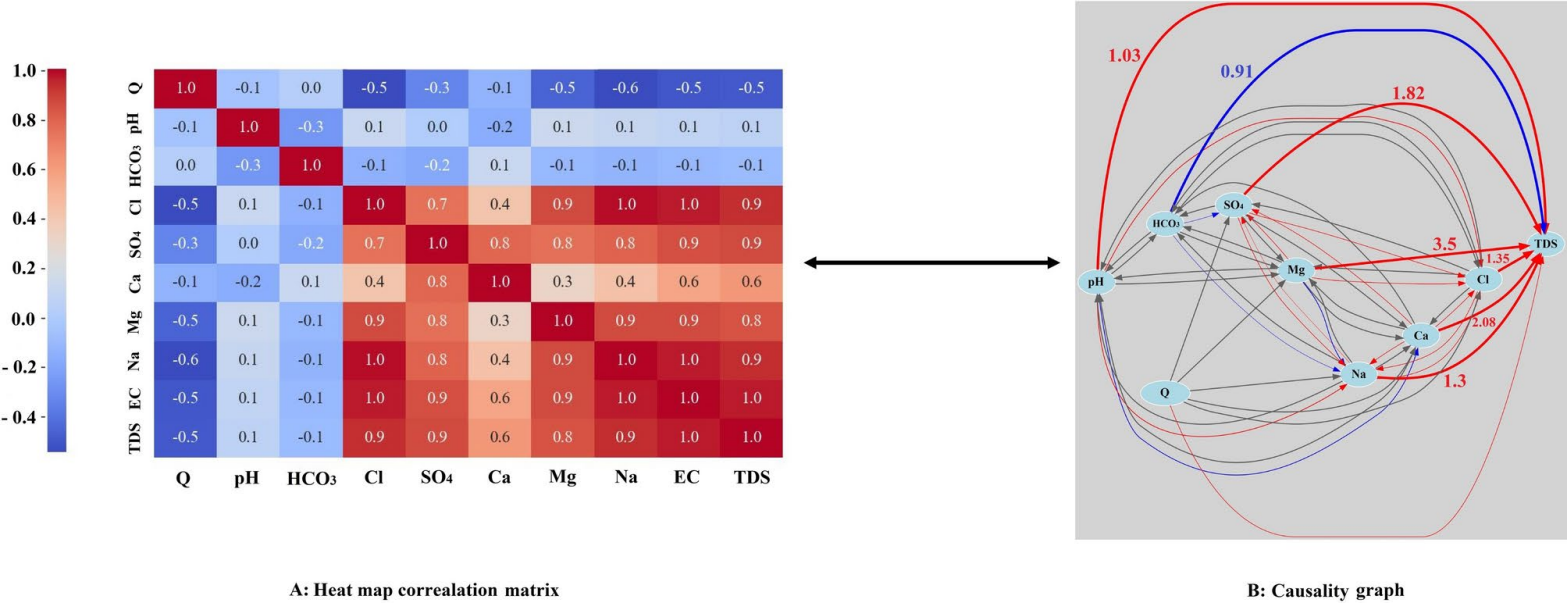
Correlation heat map matrix (**A**) and causality map (**B**) in the Hamidiyeh station.

Figure 9 shows the correlation map of quantitative and qualitative variables, along with the causality graph at Hamidiyeh station. According to this figure, Na (+ 0.9), Cl (+ 0.9), SO_4_ (+ 0.9), Mg (+ 0.8), and Ca (+ 0.6) exhibit the highest positive correlations with TDS. Conversely, Q (− 0.5), pH (− 0.1), and HCO_3_ (− 0.1) show the highest negative correlations with TDS. The quantitative values of variables with positive correlations are significantly larger than those with negative correlations. The causality graph further indicates that Mg (+ 3.5), Ca (+ 2.08), SO_4_ (+ 1.82), Cl (+ 1.35), Na (+ 1.3), and pH (+ 1.03) have the most positive effects on TDS, whereas HCO_3_ (− 0.91) is the only variable with a negative impact on TDS. It is important to note that, in this station, unlike the previous two stations, pH has a positive impact on TDS. To better illustrate the difference between correlation and causality, we refer to some examples based on Fig. 9. The correlation between Ca and Na (and vice versa) is + 0.4. Ca has a stronger positive effect on Na, whereas Na has a lesser effect on Ca. Additionally, pH has a greater positive influence on Cl than Cl has on pH, even though the correlation for both conditions (pH on Cl and Cl on pH) is + 0.1. HCO_3_ and Na exhibit a small inverse correlation with R^2^ = − 0.1. The causality graph indicates that HCO_3_ has a negative and more significant effect on Na, but the reverse is not true. Mg and Ca have a slight bilateral effect on each other, with a correlation of + 0.3. Q shows an inverse correlation with all qualitative variables. In terms of influence, Q has a slight effect on SO_4_, Mg, Na, Ca, and Cl, a positive effect on TDS, no effect on pH and HCO3, and it is not influenced by any variable.

**Fig. 10:**
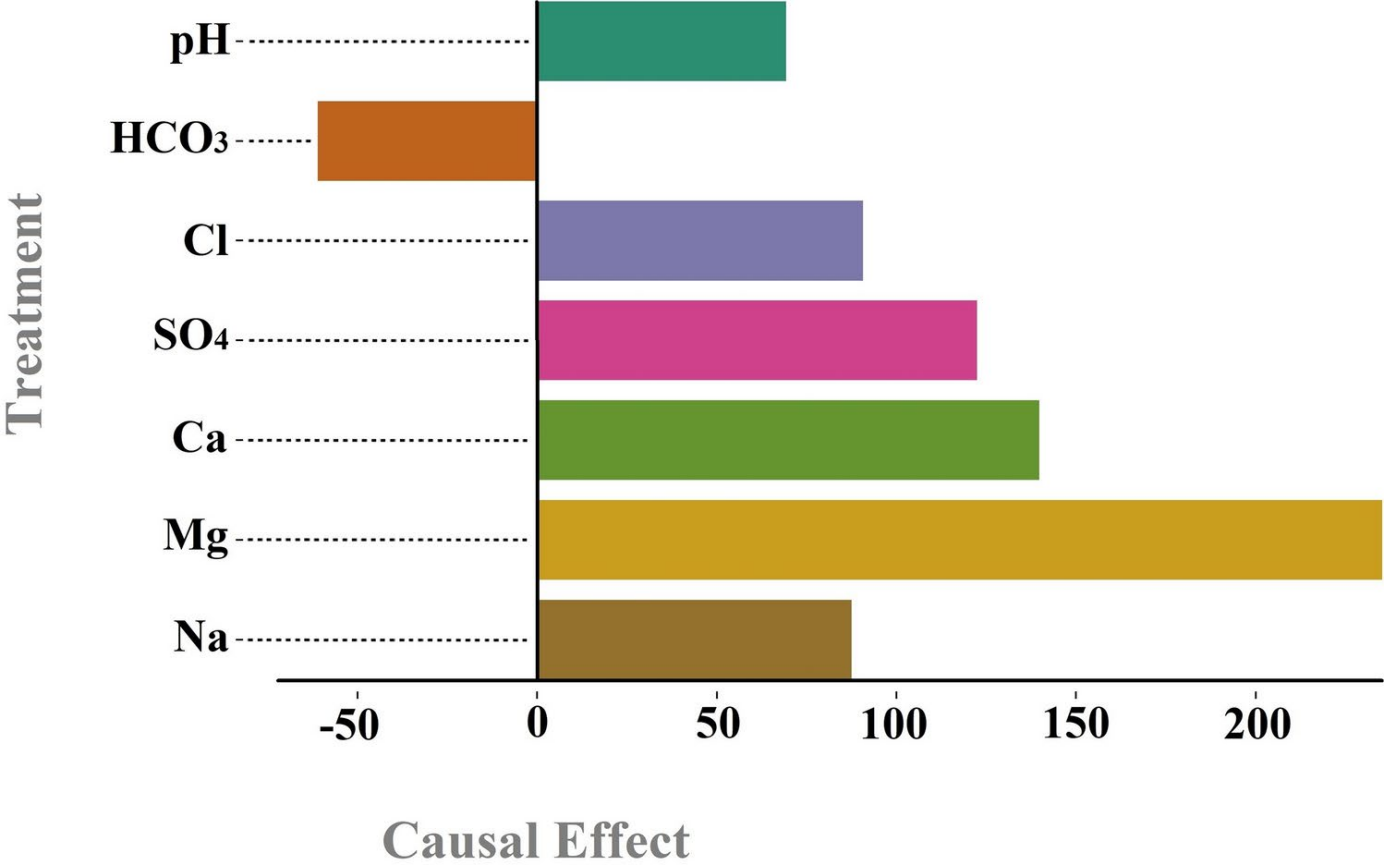
The influence of the examined variables on TDS in the Hamidiyeh station.

Figure 10 illustrates the impact of qualitative variables on TDS. According to this figure, Mg, Ca, SO_4_, Cl, Na, and pH have a progressively positive effect on TDS, while HCO_3_ is the sole variable exerting a negative influence. The bar charts for all stations (Figs. 6, 8, and 10) show the real values of causal effect. Another statistical factor that is very crucial for such study is *P*-value, that should be less than 0.05. For the whole causal effects analysis, only treatment influences with *P*-value less than 0.05 were considered. The present results confirmed the findings about the causal effect by not-only the causal value which is important but also with the statistical factor of *P*-value. Also, the causal effects have been computed with confidence intervals which enables researchers to understand the lower and upper confidence intervals for future studies. Mohammadi et al.^49^ and Zavareh et al.^48^ supported the causal links between water quality variables (e.g., TDS, EC, and ions) and hydrological factors. They showed the existing causal pathway in Na, Mg, Cl on the TDS level. It was essential to make sure the results are consistent with the experimental and empirical studies.

### Limitations of the study and future works

This present work should be seen as a first systematic causal inference model for water quality in rivers investigating the causal effect of the measured environmental variables on the TDS, with limitations that were mainly related to i) the scarcity of available data, ii) the simplifications of the adopted modelling approach. This study was a try for understanding the causal relationship of the variables and TDS without overcomplicating the analysis at this stage. A key limitation of this work was Temporal Dynamics in the analysis, in the sense that the model does not explicitly consider the temporal dynamics of the system. The simplified assumption was putting i) Time lagged effects, ii) Temporal feedback loops, iii) Time-varying confounders, aside and just focused on the causal effect under a controlled set of assumptions. This work is positioned as the foundation for the future studies that model is built toward temporal dynamics and exploring non-stationarity. The potential future work would be using advanced non-linear causal models that also consider temporal dynamics and incorporating the signal analysis. Unlike the predictive modeling, we can incorporate the socio-economic data in “Casual models” which makes a significant contribution for policy making. This helps decision makers to go beyond the conventional prediction of the future of the water quality using sensors and indicators. It is going to be a platform in which other fields can enhance the mitigation strategies that is designated by environmental engineers.

### Policy recommendations

**Fig. 11:**
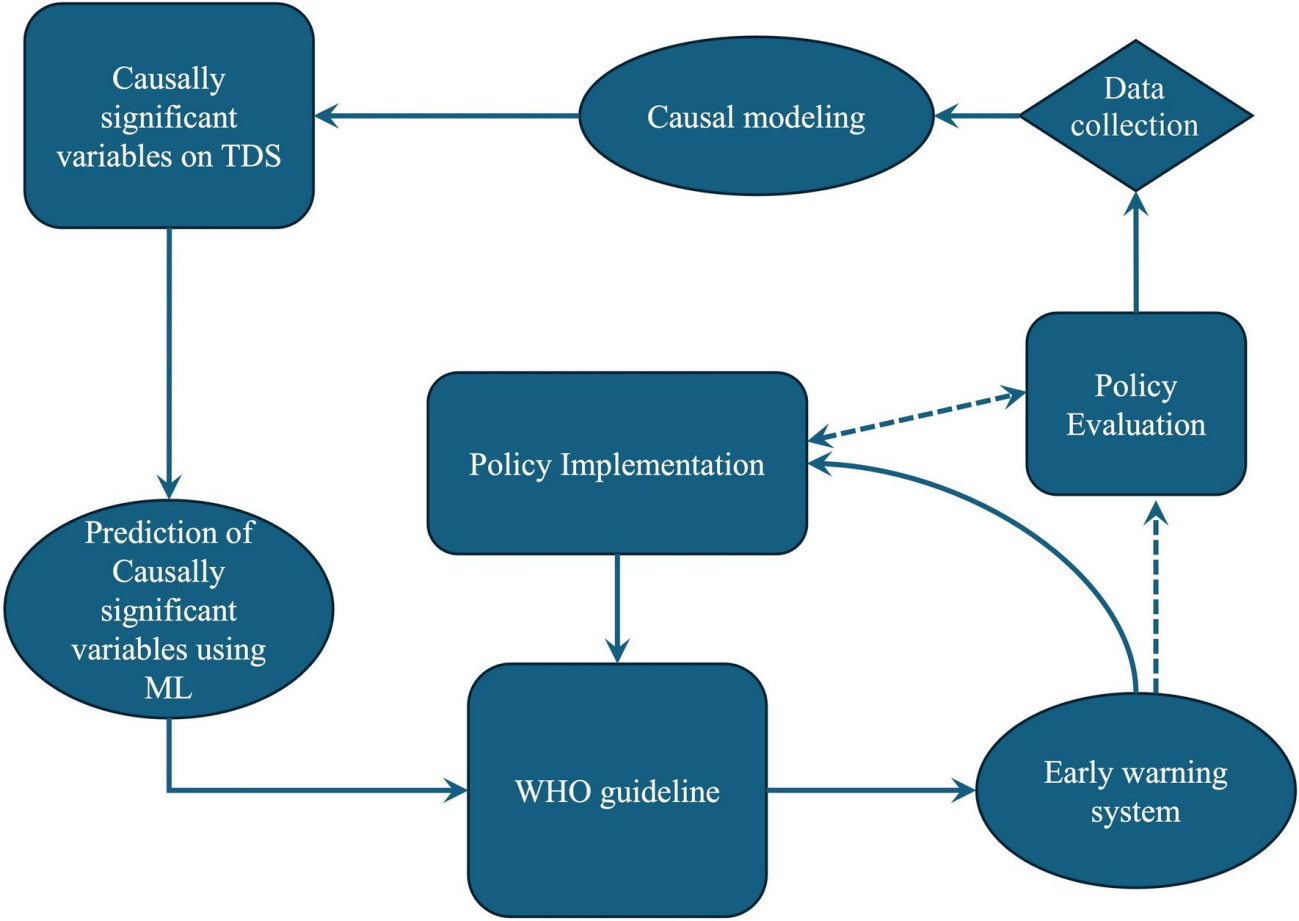
Policy making diagram.

Based on the identified causal relationships between TDS and water quality variables in the Karkheh River, several targeted policy recommendations can be made to effectively manage water quality. First and foremost, the policy makers should prioritize the key variables impacting the TDS level. Policies should focus on monitoring and regulating anthropogenic activities such as agricultural return flows and industrial waste waters, which are significant sources of Mg, Na and Cl. Also, efforts should be directed at reducing Mg and SO_4_ contributions through improved land-use practices, particularly in agricultural zones where fertilizers contribute to these ions^47,50^. As a mitigation strategy, an early warning system should be designed using predictive modeling based on WHO guidelines (with the priority of key variables) to forecast the level of the importance variables in the future then river is restored through avoiding flowing more waste material in the mainstream. As it is shown in below diagram (Fig. 11), the flow of mitigation strategies starts from policy evaluation which is highly dependent on the current status of the riverine system. To this end, causal model is essential to be employed using all available variables including climatic data and socio-economic data. After causally understanding about the significant variables, and ML algorithms are used to predict the future of the variables. This is checked using WHO guidelines and then early warning system that has been designed for alerting the variable fluctuation. It is important to frequently evaluate and enhance and develop the policy conditions using the results of the predictions and guidelines (Disclaimer: The shapes in diagram of Fig. 11 do not follow algorithmic structures so the rectangular or diamonds do not follow the algorithmic definitions).

The Karkheh River, located in a semi-arid region of Iran, is characterized by high seasonal variability in water flow, significant anthropogenic influences, and a complex interplay of hydro-chemical processes. These features make it comparable to rivers in similar climatic zones, such as other Middle Eastern and Central Asian rivers. Studies on comparable water bodies, such as the Karun River in Iran^30^ and the Cauvery River in India^50^, suggest that causal relationships, particularly the influence of cations like Mg and Na, on TDS are consistent across semi-arid and arid environments. For rivers in such regions, the results of this study can provide a robust framework for predicting and managing water quality. The effectiveness of ML-based causal inference approaches demonstrated in this study could be extended to other datasets with similar temporal and spatial resolution.

While the causal relationships identified in the Karkheh River study can be generalized to similar semi-arid rivers, caution is necessary when applying these findings to systems with differing climatic, geological, or anthropogenic characteristics. Future research should focus on validating these relationships across diverse environments and incorporating dynamic factors like climate change and extreme events to enhance the robustness of generalizations.

## Conclusion

This study represents a pioneering effort to unravel the causal relationships among water quality variables in the Karkheh River using ML techniques. By analyzing a robust dataset spanning five decades, the research provides valuable insights into the factors influencing TDS, a critical indicator of water quality. The results reveal that Mg, Na, Ca, Cl, and SO_4_ significantly contribute to TDS levels (positive influence), while HCO_3_ and pH exert negative effects. This is different to what is obtained through conventional prediction modeling which solely predict the label data without taking step further than the checking the correlation.

The application of ML-driven causal inference offers a cost-effective and scalable alternative to traditional experimental approaches, reducing reliance on resource-intensive laboratory studies. This methodological innovation not only enhances our understanding of water quality dynamics but also equips policymakers and water managers with actionable insights. Targeted interventions, such as regulating Mg, Na and Cl inputs, optimizing irrigation practices, and implementing ion-selective treatment systems, can effectively mitigate TDS-related water quality issues.

Moreover, the study’s findings pave the way for the development of early warning systems, integrating real-time monitoring and predictive modeling to proactively address water quality challenges. While the insights gained are highly relevant to the Karkheh River, they also hold potential for adaptation in other semi-arid and arid regions with similar hydrological characteristics.

Future research should explore the application of these methods to other water bodies with diverse climatic and geological conditions and incorporating dynamic factors like climate change and extreme events, enhancing the generalizability of findings. By bridging the gap between data-driven causal analysis and practical water management strategies, this study contributes to the broader goal of achieving sustainable water resource management in a rapidly changing world.

## Data Availability

The datasets generated during and/or analyzed during the current study are available from the corresponding author on request.
